# Effectiveness of alternative measures to reduce antimicrobial usage in pig production in four European countries

**DOI:** 10.1186/s40813-020-0145-6

**Published:** 2020-03-02

**Authors:** Svenja Raasch, Lucie Collineau, Merel Postma, Annette Backhans, Marie Sjölund, Catherine Belloc, Ulf Emanuelson, Elisabeth grosse Beilage, Katharina Stärk, Jeroen Dewulf, on the behalf of the MINAPIG Consortium

**Affiliations:** 1 University of Veterinary Medicine Hannover, Field Station for Epidemiology, Bakum, Germany; 2 SAFOSO, Waldeggstrasse 1, 3097 Liebefeld, Switzerland; 3 BIOEPAR, INRA, Oniris, BP40706, F-44307 Nantes Cedex 3, France; 4 Veterinary Epidemiology Unit, Department of Reproduction, Obstetrics and Herd Health, Faculty of Veterinary Medicine, Ghent University, Ghent, Belgium; 5 Department of Clinical Sciences, Swedish University of Agriculture, Uppsala, Sweden; 6 Department of Animal Health and Antimicrobial Strategies, National Veterinary Institute, Uppsala, Sweden

**Keywords:** Alternative measures, Antimicrobial consumption, Intervention study, Disease incidence, Treatment incidence, Pig

## Abstract

**Background:**

The reduction of antimicrobial usage (AMU) is in the focus in modern pig production. The objective of this study was to assess the effectiveness of alternatives to reduce AMU at herd level. In a prospective study, 68 farrow-to-finish pig herds located in Belgium, France, Germany and Sweden were recruited on a voluntary basis to implement tailor-made intervention plans to reduce their AMU. Alternative measures included improvement of biosecurity (*n* = 29 herds), vaccination (*n* = 30), changes of feeding schemes or drinking water quality (*n* = 45), improved pig health and welfare care (*n* = 21) as well as changes in stable climate and zootechnical measures (*n* = 14). Herds were followed for 1 year after implementation of measures. Annual antimicrobial expenditures or treatment records, as well as disease incidence scores were collected and compared to those of the year before intervention. AMU was measured as the treatment incidence and calculated by age category, antimicrobial class and administration route.

**Results:**

Compliance with the intervention plans was high (median 93%). AMU was significantly reduced following the implementation of alternative measures: in the median herd of the four countries, pigs were treated before intervention 25% of their expected lifespan (200 days from birth to slaughter) and after intervention 16%. AMU of suckling and weaned pigs were significantly reduced by 37 and 54%, respectively. The usage of polymyxins and tetracyclines was significantly reduced by 69 and 49%, respectively. AMU via feed and water, as well as parenteral AMU were significantly reduced by 46 and 36%, respectively. Herds with a higher AMU level before intervention achieved a bigger reduction. The majority of disease incidence were similar before and after intervention, with a few exceptions of disorders related to the gastro-intestinal tract in suckling pigs (decreased) and in breeding pigs (increased).

**Conclusion:**

Following tailor-made implementation of alternative measures, a substantial reduction of AMU in pig production was achievable without jeopardizing animal health. The AMU reduction in the youngest age categories (suckling and weaned pigs) and the reduction of group treatments via feed and water was in line with the recent European Guidelines on the prudent use of antimicrobials in veterinary medicine.

## Background

To combat antimicrobial resistance (AMR) is a priority for the European Commission [1]. Monitoring AMR and AMU on national and regional level is of great importance to create a comprehensive and reliable overview of the development and spread of AMR. Several studies identified clear associations between AMU and AMR in both human and veterinary medicine [2–6]. In livestock production, pig husbandry is one of the main sectors using antimicrobials [7–9]. An abrupt removal or restriction of antimicrobials may have negative consequences on animal health and welfare. After banning antimicrobial growth promotor (AGP) use (1998–1999) in Denmark farmers experienced a short-term increased incidence of gastrointestinal disorders in weaned pigs [10]. The unintended consequences from antimicrobial use restrictions for disease treatment or prevention have been assessed by several studies. Overall, the results indicated, that the consequences were temporary and minor [11–13]. Yet, further research is needed to assess the impact on animal health and welfare from antimicrobial use restrictions and how these effects could be mitigated by improvements of animal health, management and housing. Thus, disease preventing strategies are required to reduce AMU without subsequent increases in disease frequencies. Whilst ensuring economic viability and animal health, the demand on effectiveness and practical feasibility of alternatives to antimicrobials is high. Many alternatives have been described in literature, but little is known about their effectiveness. The study by Postma et al. 2015 [14] provided a list of possible alternatives, which has been ranked by pig health experts according to their expected effectiveness, feasibility and return on investment. Most favoured alternatives were the improvement of biosecurity measures, intensified vaccination, the use of zinc/metals in feed, the improvement of feed quality, the use of regular diagnostic testing and a clear herd specific action plan based on diagnostics and historical data. EFSA (European Food Safety Authority) and EMA (European Medicines Agency) released a Joint Scientific Opinion on measures to reduce the need of antimicrobial treatments in animal husbandry in the EU and the resulting impacts on AMR [15]. Even though national reduction strategies have been successfully implemented in some Member States and several alternatives to antimicrobials have been studied, there is still a gap of knowledge in relation to their effectiveness under field conditions. Moreover, the multiplicity of factors contributing to AMU makes it difficult to quantify the impact of a single alternative measure on the AMU.

This publication was a result of data collected during the MINAPIG project. The project was conducted to evaluate strategies for raising pigs with minimal AMU in four EU countries (Belgium, France, Germany and Sweden). The objective of this study was to assess the impact of herd-specific measures and associated compliance level on the AMU level in farrow-to-finish farms. More specifically, the aim was to explore the following questions: what AMU reduction can be achieved i) by age group, ii) by antimicrobial class and iii) by administration route. Moreover, we investigated the impact of the implementation of AMU-reducing measures on pig health.

## Materials and methods

### Study design

**Fig. 1:**
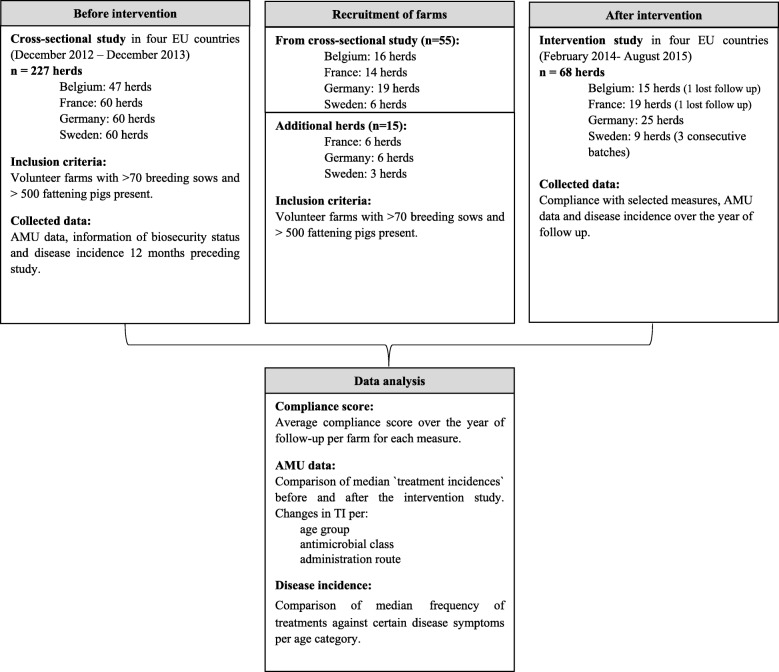
Overview of the study setup

Between February 2014 and August 2015 an intervention study was conducted in 68 farrow-to-finish farms in four EU countries. Farms were located in Belgium (*n* = 15), France (*n* = 19), Germany (*n* = 25) and Sweden (*n* = 9). Prior to the intervention study a cross-sectional study had already been conducted in these countries [16–18]. The focus of the cross-sectional study was to explore the relationship between AMU and farm management characteristics, biosecurity practices and health status, as well as farmer’s attitude and behaviour towards AMU. In order to investigate the efficacy of the implementation of alternative measures, AMU data from the cross-sectional study and the intervention study were compared. Each farm provided data on biosecurity measures, disease incidence and antimicrobial expenditures or detailed treatment records during a period of 12 months preceding (antimicrobial usage data for France during the cross-sectional study was only available for the last produced batch) and 12 months following the initiation of the intervention study. For the Swedish farms treatment records were only collected for three consecutive batches. Each farm served as its own control (Fig. 1).

### Recruitment of farms

Farms were primarily recruited among those who participated in the cross-sectional study (Fig. 1). Inclusion criteria were defined and only herds with at least 70 sows and 500 fattening pigs produced per year participated in this study. A total of 68 herds (Belgium *n* = 15, France *n* = 19, Germany *n* = 25 and Sweden *n* = 9 herds) agreed to participate in the intervention study (more details on the recruitment procedure are presented in Additional file 1). In Germany and Sweden, farmers received financial compensation for collecting and providing data, 200€ and 1300€, respectively.

### Data collection

Data from the cross-sectional study was available for 16 Belgian, 14 French, 19 German and six Swedish farms. Details of the data collection in farms in the cross sectional study was described in Sjölund et al. [17]. Briefly, data on AMU of the participating herds was collected for one year preceding the herd visit in Belgium, Germany and Sweden and for the last batch produced in France. Information on AMU originated from invoices from prescribing herd veterinarians or feed companies, treatment records or from direct interview with the farmer. Since 15 farms (additional farms: France *n* = 6; Germany n = 6; Sweden *n* = 3) did not participate in the cross-sectional study and were recruited specifically for the intervention study, these farms were asked to provide data on AMU and disease incidence from the year preceding the study (Fig. 1). During the intervention study data on disease incidence and AMU was collected for one year in Belgium, France and Germany. Herd veterinarians in Belgium and France provided receipts of all antimicrobial expenditures of the farm during the year of follow-up. In Germany either the herd veterinarian or farmer provided copies of the obligatory application and dispensing records. Swedish veterinarians retrieved the AMU data from herd treatment records as described by Sjölund et al. (2015) for three consecutive farrowing batches. For sows, AMU was only collected during the nursing period [19]. The information on the receipts and records included commercial product names, amount and size of the antimicrobial packages. In Belgium and France all prescribed antimicrobials were re-allocated by the farmer to a given animal category, namely suckling pigs, weaned pigs, fattening pigs or breeding pigs (i.e. sows, gilts and boars). In Sweden this was retrieved from the treatment records and in Germany the dispensing and application form provided information on the treated animal category. To define a population at risk to receive antimicrobial treatments, the average number of present pigs per age category (i.e. suckling pigs, weaned pigs, fattening pigs and breeding pigs) was collected from the farm management system or by interviewing the farmer. To harmonize and improve data quality across the participating countries all collected data was entered in a Microsoft Office Access© (version 2010) database.

### Herd specific intervention plan and compliance

In Belgium, France and Germany, an initial herd visit, together with the farmer, the herd veterinarian and the project researcher was organized to define a herd specific intervention plan. In Sweden the herds were either visited initially by the herd veterinarian of the G&D or by one of the two project researchers. Data and results from the cross-sectional study were used as a basis for discussion about options to reduce AMU. The alternatives were summarized in six general categories for improvement namely i) external biosecurity status (measures to prevent pathogens from entering into herd premises or a group of animals), ii) internal biosecurity status (measures to prevent the spreading of pathogens within herd premises or a group of animals), iii) herd vaccination scheme, iv) feed or water quality or composition, v) pig health care (management/focus on individual, vi) pig stable climate and other zootechnical measures. In order to specify the alternative measures each category was divided in predefined options (Table 1). The most feasible and promising options to reduce AMU regarding the herd health problems were considered by the farmer and veterinarian. Subsequently, they decided on type and number of options to implement. Participating herds were visited at least twice and up to a maximum of six times by a project researcher or the herd veterinarian (for some of the Swedish herds). During the visits, all issues related to the implemented measures were discussed. If herd health or individual pig health was jeopardized, the intervention was adjusted. Intermediate phone calls with the herd veterinarian and farmer were conducted to follow the development of the implemented measures. To describe the compliance with the predefined measures an average compliance score over the year of follow-up per farm was computed for each measure. On every follow up visit, farmers had to report on a scale from 1 (=no attempt to implement the measure) to 5 (=perfect implementation), whether they followed the predefined intervention plan. Scores were then converted into an average percentage of compliance to the intervention plan (i.e. total farm score × 100 / maximum achievable score). Moreover, herd veterinarians and the project researcher commented on the observed compliance. Their reports corresponded to the score given by the farmer, so no adjustments were made to the compliance reported by the farmers.

### Disease incidence

For information on the disease incidence, farmers were asked about the average frequency of treatments (e.g. antimicrobials, electrolytes, probiotics etc.) against certain disease symptoms per age category over the preceding year. Annual data on the disease incidence from both the cross-sectional study and the intervention study was available in 15 Belgian, 13 French and 20 German farms. In Sweden data on diseases was collected batch-wise and expressed as a percentage. Thus, the Swedish data was incomparable with the data from the other countries and not included in the analysis. In the intervention study, farmers were asked to provide these data by the end of the study. Median disease incidence scores before and after the intervention study were compared for each age category.

### Quantification of AMU

For the detailed analysis of the AMU, antimicrobial treatment data were converted into a `treatment incidence` (TI). The TI expresses the number of animals out of a 1000 being treated with a daily dose of antimicrobials or, when divided by 10, the percentage of their life expectancy they are treated with one daily dose of antimicrobials [20]. Previously developed defined daily doses animal (DDDA’s) harmonized for the MINAPIG project were used for calculating the TI and originated from a consensus DDDA list previously published [21]. The period at risk for each animal category was defined as the time period a pig could receive an antimicrobial treatment. For breeding animals, it was set to 365 days, for suckling, weaned and fattening pigs the rearing periods of the individual herds were used. Since the Swedish herds provided data from three consecutive batches the risk period for the breeding sows was adapted accordingly. This means that the period at risk for a “batch” of sows was set to 158.7 days (365 days divided by 2.3, the average number of farrowings per sow per year [22]). In order to enhance comparability between farms, harmonized weights for the animal categories (in kilogrammes) were defined, namely: two kg for suckling pigs, seven kg for weaned pigs, 35 kg for fattening pigs, 60 kg for gilts and 220 kg for sows [17]. Moreover, the TI of suckling, weaned and fattening pigs were combined into a `TI 200 days` (TI200d), assuming an expected lifespan of 200 days. Hence, the standardised TI200d corrected for possible differences in ages at slaughter between the participating farms (for details see Sjölund et al. (2016) [17]). Furthermore, a TI for each antimicrobial class, namely: third generation cephalosporines, aminoglycosides, aminopenicillins, benzylpenicillins, benzylpenicillins-combinations, florfenicols, fluorquinolones, macrolides, macrolide-combinations, polymyxins, pleuromutilins, tetracyclines and trimethoprim-sulfonamides, and administration route (oral and parenteral) was computed for every farm. Treatment incidences of topical products were excluded from the analysis because they represented a negligible part of AMU.

### Data processing and statistical analysis

For the purpose of analysing the effect of different alternative measures, median TIs and median disease incidence scores before and after the intervention study were compared using non-parametric Wilcoxon signed-rank testing. A *p*-value of 0.05 was used as a significance threshold. Spearman rank correlation was used to test for associations between treatment incidence scores before and after intervention. Descriptive and analytical statistics were performed using the open-source environment R 3.0.2 (R Core Team, 2013, www.r-project.org).

## Results

### Herd specific intervention plans and compliance

A total of 70 herds were enrolled in the intervention study, but two herds were lost from follow-up due to farmers’ personal issues (*n* = 1) and due to changing herd veterinarian, who was not willing to participate (*n* = 1). Changes in feed or water quality or composition (*n* = 45) was the most commonly implemented intervention (Table 1). The most common option in this category was the implementation of therapeutic zinc oxide in the feed for piglets (*n* = 18). In 11 Belgian herds zinc oxide was added to the post-weaning feed at 2500 ppm for 10 to 14 days. This option was only possible due to a change in legislation shortly before the start of the study (September 2013), which authorized the therapeutic use of zinc oxide [23]. In Germany, seven herds added zinc oxide at 150 ppm for seven to 14 days post-weaning instead of using a combination of colistin and zinc. The median compliance percentage for the last-mentioned category was the highest (100%), whereas the compliance for improvement options in the category ‘external biosecurity’ was the lowest (73%) (Table 1).

**Table 1:** Distribution of alternative measures in the participating herds (*n* = 68) and median compliance percentage per category

General categories for improvement (number of farms)	Options for improvement (number of included measures)^a^	Median compliance percentage in general category (Min-Max)
External biosecurity (*n* = 9)	Purchasing policy/gilts acclimatisation (*n* = 8)	73 (20–100)
	Removing of animal carcasses (*n* = 2)	
	Vermin control (*n* = 1)	
Internal biosecurity (*n* = 20)	Suckling period management (n = 9)	75 (0–100)
	Farm compartmentalising, working lines (n = 6)	
	Cleaning and disinfection (*n* = 6)	
Vaccination scheme (*n* = 30)	Altering of existing vaccination protocols (*n* = 5)	88 (0–100)
	Implementation of a new vaccination (*n* = 29)	
Feed or water quality or composition (*n* = 45)	Feed additives zinc/metal (n = 18)	87 (0–100)
	Feed scheme revision (*n* = 10)	
	Feed and water acidification (*n* = 12)	
	Cleaning and disinfection of water pipes (*n* = 7)	
	Feed additives phytotherapy and other additives (*n* = 14)	
	Feed quality improvement (e.g. change in fat, protein or fibre content) (*n* = 5)	
	Feed additives pre- and probiotics (*n* = 4)	
	Water quality control (*n* = 3)	
Pig health care (*n* = 21)	Increased diagnostics (*n* = 7)	89 (0–100)
	Alternative treatments protocols in case of symptoms (e.g. with anti-inflammatory products or prostaglandins) (*n* = 5)	
	Revision of deworming scheme (*n* = 4)	
	Stopping surgical castration (n = 3)	
	Hospital pens put in place (*n* = 3)	
	Euthanasia of runt suckling piglets (n = 1)	
Pig stable climate and other zootechnical measures (*n* = 14)	Climate adjustments (*n* = 7)	100 (20–100)
	Change of genetics (*n* = 2)	
	Animal transfer adjusted (avoidance of re-mixing of piglets or having pens with heterogeneous pigs (n = 4)	
	Building renovations (*n* = 3)	
	Reduced pig density (*n* = 2)	
	Farrowing processed slowed down (*n* = 2)	

^a^The number of included options is higher than the number of participating farms, because farmers and herd veterinarians decided to implement one or multiple options from one or several categories

### Disease incidence score before and after intervention

For the comparison of median disease incidence scores before and after the intervention study, a total of 48 herds were considered in the analysis (Belgium *n* = 15; France *n* = 13; Germany *n* = 20) (Table 2). Treatment frequencies of breeding pigs against disorders of the central nervous system and gastrointestinal tract increased during the intervention study (*p* <  0.001), whereas treatments against skin diseases (*p* = 0.04) and udder diseases decreased (*p* = 0.03). Suckling pigs were more frequently treated against disorders of the central nervous system in the intervention study (p <  0.001), but treatments against disorders of the gastrointestinal tract decreased (p <  0.001). Treatment frequency of weaned pigs against disorders of the locomotor system decreased during the intervention study (*p* = 0.01). Fattening pigs were more frequently treated against disorders of the gastrointestinal tract during the intervention study (*p* = 0.02) (Table 2).

**Table 2:** Comparison of median disease incidence scores^a^ per age categories before and after intervention (*n* = 48)

Age category	Disorders of	Median before intervention (Q25; Q75^b^)	Median after intervention (Q25; Q75)	*p*-value^c^
Suckling pigs	locomotor system	3 (2; 4)	3 (2; 3)	0.36
	gastro-intestinal tract system	3 (2; 4)	1 (1; 2)	**< 0.001**
	respiratory tract system	1 (1; 2)	1 (1; 2)	0.55
	central nervous system	1 (1; 2)	2 (2; 3)	**< 0.001**
	skin	1 (1; 2)	1 (1; 2)	0.92
Weaned pigs	locomotor system	3 (2; 4)	3 (2; 3)	**0.01**
	gastro-intestinal tract system	2 (1; 4)	2 (2; 3)	0.19
	respiratory tract system	2 (1; 3)	2 (1; 3)	0.97
	central nervous system	2 (2; 3)	3 (2; 4)	0.24
	skin	2 (1; 2)	2 (2; 3)	0.18
Fattening pigs	locomotor system	2 (2; 3)	2 (2; 3)	0.45
	gastro-intestinal tract system	1 (1; 2)	2 (2; 3)	**0.02**
	respiratory tract system	2 (1; 3)	2 (1; 3)	0.84
	central nervous system	1 (1; 2)	1 (1; 2)	0.11
	skin	1 (1; 2)	1 (1; 2)	0.08
Breeding pigs	locomotor system	2 (2; 3)	3 (2; 3)	0.70
	gastro-intestinal tract system	1 (1; 1)	1 (1; 2)	**< 0.001**
	respiratory tract system	1 (1; 2)	2 (1; 2)	0.60
	central nervous system	1 (1; 1)	1 (1; 1)	**0.02**
	skin	1 (1; 2)	1 (1; 1)	**0.04**
	reproductive tract system	2 (2; 3)	2 (2; 3)	0.80
	udder	2 (2; 3)	2 (2; 3)	**0.03**

^a^Scores are composed of a five-point Likert scale (1 = never, 2 = rarely; 3 = occasionally; 4 = regularly; 5 = commonly/always). Significant (*p* < 0.05) results are highlighted in bold

^b^25th and 75the quartiles

^c^Wilcoxon signed rank test (*n* = 48 farrow-to-finish herds)

### Treatment incidences per age category before and after intervention

For the analysis of the AMU before and after the intervention study, 14 Belgian, 19 French, 25 German and nine Swedish herds were included. One Belgian farm was excluded from the analysis due to un-reliable data. Median TIs in the different age categories, antimicrobial classes and administration routes differed between and within countries (see Additional file 2). Following the implementation of interventions, AMU was significantly reduced. In the median herd of the four countries, pigs were treated during 25% of the expected lifespan before the intervention (TI200d: 247.3) and during 16% of the expected lifespan after the intervention (TI200d: 160.2) (*p* < 0.001), which is a reduction of 35% (Table 3). Moreover, treatment incidences of suckling and weaned pigs were significantly (*p* < 0.001) reduced by 37 and 54%, respectively (Table 3). In the fattening and breeding pigs, no significant changes were observed.

**Table 3:** Comparison of median treatment incidences (TI) for different age categories before and after intervention (*n* = 67)

Parameter	Median before intervention (Q25; Q75)	Median after intervention (Q25; Q75)	*p*-value^a^	Difference in percentage (%)
TI suckling pigs	279.9 (158.0; 484.6)	176.2 (80.2; 391.7)	**< 0.001**	− 37
TI weaned pigs	568.0 (113.1;1072.3)	261.2 (54.4; 573.1)	**< 0.001**	−54
TI fattening pigs	8.0 (0.8;37.7)	7.7 (0.8; 44.0)	0.89	−3
TI200d	247.3 (92.7; 451.8)	160.2 (59.7; 303.6)	**< 0.001**	−35
TI breeding pigs	14.0 (4.5; 32.0)	17.1 (4.2; 45.3)	0.5	+ 18

^a^ Wilcoxon signed rank test (*n* = 67 farrow-to-finish herds). Significant (*p* < 0.05) results are highlighted in bold

### Treatment incidences per antimicrobial class and administration route before and after intervention

The usage of some critically important antimicrobials for human medicine (i.e. fluoroquinolones and 3rd generation cephalosporins) was reduced significantly (Table 4), especially in Belgium (TI for 3rd generation cephalosporins before: 83.9; after: 0.7) (see Additional file 2). In the median herd of the four countries, TIs of polymyxins (critically important antimicrobial for human medicine) and tetracyclines were significantly reduced (TI polymyxins: *p* < 0.001; TI tetracyclines: *p* = 0.01) with a median of 106.8 and 50.7 before the interventions and 33.2 (69% reduction) and 26.1 (49% reduction) after the interventions (Table 4), respectively. TIs for macrolides decreased in France, Germany and Sweden, whereas the usage increased in Belgium (before: 2.5; after: 27.5) (see Additional file 2). Usage of benzylpenicillin-combinations was significantly (*p* = 0.01) reduced (Table 4); this was mainly influenced by Germany, where the maximum TI before the intervention was 9.9 and after the intervention 0.0 (see Additional file 2). Median treatment incidences over the four countries for feed/water and parenteral administrations were significantly reduced by 46% and 36% after implementation of interventions (TI feed-water: *p* < 0.001; TI parenteral: *p* = 0.01) (Table 4).

**Table 4:** Comparison of median treatment incidences (TI) for different antimicrobial classes and administration route before and after intervention (*n* = 67)

Parameter	Median before intervention (Q25; Q75)	Median after intervention (Q25; Q75)	p-value^a^	Difference in percentage (%)
TI 3rd generation cephalosporins	0 (0.0; 10.3)	0 (0.0; 1.1)	0.09	0
TI Aminoglycosides	0 (0.0; 0.7)	0 (0.0; 0.6)	0.24	0
TI Aminopenicillins	139.6 (20.2; 414.0)	136.0 (15.9; 350.3)	0.19	−3
TI Benzylpenicillin	0 (0.0; 14.5)	0 (0.0; 17.8)	0.62	0
TI Benzylpenicillin-combinations	0 (0.0; 14.5)	0 (0.0; 5.6)	**0.01**	0
TI Florfenicols	0 (0.0; 0.0)	0 (0.0; 0.5)	0.81	0
TI Fluorquinolones	9.5 (3.7; 27.3)	7.6 (1.0; 28.0)	0.15	−20
TI Macrolides	12.6 (0.3; 208.7)	12.4 (0.0; 203.4)	0.84	−1
TI Macrolide-combinations	0 (0.0; 0.6)	0 (0.0; 0.2)	0.12	0
TI Pleuromutilins	0 (0.0; 0.0)	0 (0.0; 0.0)	0.18	0
TI Polymyxins	106.8 (1.1; 467.8)	33.2 (0.0; 155.4)	**< 0.001**	−69
TI Tetracyclines	50.7 (0.2; 255.1)	26.1 (0.1; 107.1)	**0.01**	−49
TI Trimethoprim-Sulfonamides	2.3 (0.0; 30.4)	0.1 (0.0; 22.8)	0.60	− 96
Administration route parenteral	300.0 (141.2; 493.4)	192.5 (111.7; 406.3)	**0.01**	−36
Administration route feed/water	601.3 (69.8; 1295.7)	322.0 (77.4; 632.2)	**< 0.001**	−46

^a^Wilcoxon signed rank test (n = 67 farrow-to-finish herds). Significant (*p* < 0.05) results are highlighted in bold

### Correlation of the achieved TI200d reduction

**Fig. 2:**
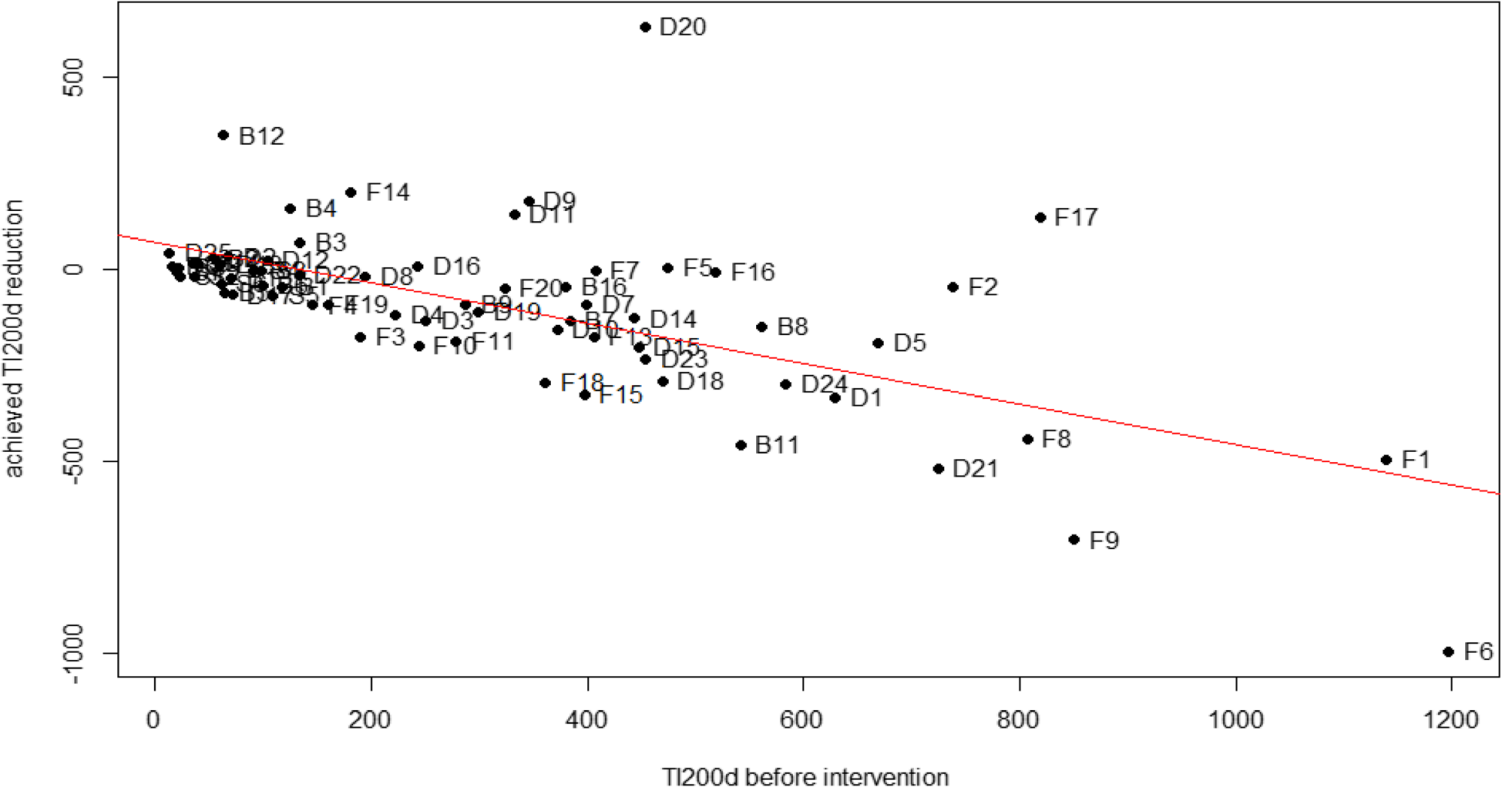
Correlation of the achieved TI200d reduction. Herds with a higher AMU before intervention achieved a larger reduction. Legend: Correlation between achieved reduction of the treatment incidence of growing pigs from birth to slaughter with a standardised life span of 200 days (TI200d after-TI200d before) and treatment incidence before (TI200d before) intervention in Belgian (*n* = 14), French (*n* = 20), German (*n* = 25) and Swedish (*n* = 9) farrow-to-finish pig herds. (Spearman rank correlation coefficient ρ = − 0.53)

Figure 2 illustrates the correlation between the change in TI200d (TI200d after-TI200d before = TI200d reduction) and the TI200d before intervention in the participating herds. Herds with a higher AMU before intervention achieved a larger reduction (Spearman rank correlation coefficient ρ = 0.74). In some herds the AMU increased after implementing interventions (17/67) while other herds managed a substantial reduction (50/67) (Fig. 2).

## Discussion

### Interventions and compliance

In this study the suggested interventions to reduce AMU were inspired by herd specific problems and diagnostic results each herd experienced by the time of the visits. It was hypothesized that implementing a predefined universal intervention plan for all participating herds would have led to a poor compliance and a poor effect on AMU as some interventions would have been unnecessary. The significant reduction of AMU was rather the overall effect of the implemented measures and cannot be extrapolated to individual measures. Moreover, a key element of a good compliance was the coaching/guidance of the farmer throughout the follow-up year and a close cooperation between herd veterinarian, farmer and external coach, as previously suggested by Postma et al. [24]. The median compliance score of the different categories of improvement was relatively high, ranging from 73% (internal biosecurity) to 100% (pig stable climate and other zootechnical measures). The willingness of farmers to make adjustments on their farm might depend on its economic benefit. Especially implementing or altering biosecurity measures are believed to be expensive or impractical [25, 26]. Therefore, it might be easier for the farmer to implement direct changes to a feeding scheme (e.g. add zinc oxide to the post-weaning feed) rather than alter daily habits related to biosecurity (for internal biosecurity e.g. change working lines). Yet, this perception is not necessarily true since different studies clearly demonstrated, that altering biosecurity measures on farms is feasible and beneficial [16, 27]. Another barrier which influenced the compliance was the need to train farm workers, especially on larger farms. Some of the interventions required a strict adherence to work processes. Therefore, all employees needed to be encouraged and trained to harmonize their workflow. Collineau et al. (2017) showed that farms with higher compliance to the intervention measures tended to achieve bigger AMU reduction [28].

### Disease incidence

A substantial reduction of AMU on herd level was achievable without major impact on pig health. The estimated treatment frequency against predefined categories of disease symptoms before and after the intervention study was used as a proxy of disease incidence in the different age categories. It should be noted that the sample size for this analysis was small and a five-point scale is not a very discriminative parameter. Nonetheless, we observed a significant reduction in the antimicrobial usage of suckling pigs and at the same time a significant reduction of symptoms related to gastro-intestinal diseases. Since neonatal porcine diarrhoea (NPD) is a common clinical condition in new-born piglets, some of the implemented alternative measures (e.g. improved feed and water quality, optimisation colostrum supply via split-suckling management) focused on this problem [29, 30].

The treatment against diseases of the central nervous system in piglets increased. A possible explanation for this effect could be that farmers focussed on individual treatments during the interventions. Thus, they intensified the observation of sick and diseased animals and reported more treatments against diseases of the central nervous system.

During the intervention period, farmers also observed a reduction of disease symptoms related to disorders of the locomotor system in weaned pigs. Participating farms with a known history of reoccurring polyarthritis outbreaks in weaned pigs improved hygiene protocols in the farrowing and nursing unit such as intensified disinfection with an appropriate disinfectant and/or changed needles and castration blades between litters. These measures reduce the transmission of infectious agents and have been recommended as useful intervention strategies in previous studies [31]. Moreover, some farms introduced autogenous vaccines in the sows pre-farrowing in order to provide new-born piglets with maternal antibodies. Immunization of sows has been reported as an effective control strategy to reduce neurological signs, depression and microscopic lesions of meningitis due to a *Streptococcus suis* infection in weaned pigs [32]. The target in farms with reoccurring polyarthritis outbreaks in weaned pigs was to reduce the oral medication with amoxicillin for entire batches in the nursery period.

In breeding pigs, farmers observed more gastro-intestinal disorders during the intervention study. This may be linked to the introduction of the Porcine Epidemic Diarrhoea Virus (PEDV) in 2014 in Germany [33]. In fact, one German farm experienced a severe PEDV outbreak during the intervention study. As a result, farmers might have been more sensitive to the observation and treatment of gastro-intestinal symptoms like diarrhoea in the breeding pigs. This might be reflected in the TI breeding pigs in Germany which increased by 75% (before intervention: 10.3; after intervention: 41.2).

### AMU reduction

The median treatment incidence in the youngest pigs was reduced by 37% (i.e. suckling pigs), in weaned pigs by 54%, in fattening pigs by 3% and in pigs from birth till slaughter (TI200d) by 35%. The significant reduction, mainly in suckling and weaned pigs, could be explained by the fact that, among the participating countries, especially suckling and weaned pigs received most of the antimicrobial treatments before the intervention and were hence targeted in priority by the implemented alternative measures. This is in line with other studies, where especially weaned pigs receive most of antimicrobial treatments [7, 9, 17, 34, 35]. Callens et al. (2012) reported that 90% of oral group treatment was administered between birth and 10 weeks of age (farrowing and nursery period). The main indication for orally administered colistin and amoxicillin was post-weaning *E. coli* (*Escherichia coli*) infections (colistin) and preventive treatments against streptococcal infections (amoxicillin) [7]. A more recent study by Sarrazin et al. (2018) observed peaks in frequency of treatment during weeks one, four and nine of the rearing period [9]. In accordance with Sjölund et al. (2016) it is suggested, that pigs are often treated at strategic time points (castration in week one, weaning at week 4 and beginning of fattening period in week 9), when they are more prone to become infected [19]. Moreover, a higher AMU at a younger age was associated with a higher use in older pigs. Thus, the reduction of AMU should already start in the suckling period, as this seemed to affect the AMU in the consecutive weaning and fattening period [9]. A study by Diana et al. (2017) demonstrated, that rearing pigs without preventive antimicrobial treatment in the weaning period is possible without jeopardising animal health and welfare indicators, which is in line with our study [36].

Group treatments via feed or water were commonly applied before the intervention in Belgium, France and Germany. In contrast, in the Swedish pig herds there were almost no group treatments through feed and water and these herds accounted for the lowest AMU before and after the intervention. The variations between countries could be partly explained by differences in prevalence of pathogens, like Porcine Reproductive and Respiratory Syndrome Virus (PPRSV). Sweden is declared free of PRRSV and pig herds are not exposed to severe diseases and consequences concomitantly caused by a PRRSV infection. Moreover, pig and herd density may influence transmission between herds and Sweden has a low pig density compared to the other participating countries [17]. In this study participating herds included measures to avoid the treatment of entire batches and tried to focus the treatment of single individuals within a batch. Treatment of entire batches with antimicrobials via oral route increases consumption compared to medication of individual animals [17]. The results of this study showed, however, that treatment incidences for both administration routes were significantly reduced. The early parenteral treatment of diseased individual animals resulted in a reduced need of oral applied antimicrobial therapy of a whole group of animals. Removal of unnecessary use was another key element of the AMU reduction in this study (e.g. omitting the treatment of suckling pigs with benzylpenicillin after surgical castration or omitting the application of polymyxins in feed or water after weaning piglets to prevent post weaning diarrhoea (PWD)). A significant reduction of 69% was observed for polymyxins (almost entirely colistin). According to the ninth ESVAC report, the sales of polymyxins decreased by 66.4% during 2011–2017 over 31 reporting countries [37]. The more pronounced reduction in the use of polymyxins in this study is likely due to the application of zinc oxide as an alternative for colistin in the treatment of PWD in weaned pigs. Additionally, the extra interventions (e.g. changes in feed or water quality or composition) and intensive coaching enhanced the effect on a reduced use of polymyxins. Even though these additives showed to have a positive effect on the gut health in young pigs, there is an increasing concern that high doses of zinc oxide leads to an increased prevalence of AMR in bacteria in weaned pigs and cause an environmental burden [15]. Therefore, the EMA decided to ban the therapeutic use of zinc oxide in feed by 2022 at the latest [38]. This may cause an increase in AMU following the ban unless alternative measures are implemented. The observed reduction of critically important antimicrobials (CIA) especially in Belgian herds demonstrated that the application of preventive measures instead of (routine) use of prophylactic treatments with highly potent antimicrobial classes was feasible. This is in line with the recent results of the BELVET-SAC report (2018), which demonstrated a very substantial reduction in the sales of 3rd and 4th generation cephalosporins and fluoroquinolones (79.1%) between 2011 and 2018 [39]. The results showed that especially herds with a high AMU before the intervention could achieve a bigger reduction of AMU during the intervention study. All participating farms were approached with benchmarked results of the cross-sectional study. This is in line with the policy in several EU member states that already use benchmarking of antimicrobial use as a key tool to trigger reduction strategies on herd level [15]. Moreover, the willingness to implement alternative strategies might be more present when there is more need for improvement (e.g. herds with a high AMU). Countries with AMU monitoring systems successfully reduced AMU for many reasons. But one main reason might be the improved awareness in both society and agricultural sector. Consequently, the pressure to move towards a more prudent AMU was increased on farms with a high AMU. In contrast farms with a correspondingly low AMU might be hesitant to change their management practices and perceive less need to contribute towards a lower AMU in their herds.

### Study design

Since it was difficult to provide control farms to compare with intervention farms, it was decided that each farm served as its own control. The definition of the control herd was the particular limitation of the experimental design of the study. As pig farming requires a constant adjustment of management practices related to the herd health status. Finding farms not changing their practices over one year was considered impossible. Thus, using each herd as its own control was the most suitable approach in this study. For each herd there was a different intervention implemented, which was tailor-made to its specific health and production situation. This approach was used to maximize the expected efficacy of the intervention, as well as the compliance with recommended measures. Moreover, this study was performed under uncontrolled settings, and could be biased by factors external to the intervention. The comparison of selected outcome variables before and after intervention (AMU, disease incidence) was possible, because detailed data from the year proceeding the interventions study was available for each participating farm. One of the main limitations of the present study relates to the way of applying a direct comparison between groups (non-parametric comparison of medians). During the study most of the technical information, antimicrobial usage and information on the disease incidence were collected on two time points: the last batch produced before the implementation of the intervention and at the end of the study. The last batch produced, was used as the reference to evaluate the impact of the intervention. In order to improve data quality and analysis different statistical tools, such as statistical process control (SPC) could be used. But in order to perform such an analysis, a time series data collection with graphical presentation of data would be needed. This would allow more insights into the data and show an improvement of defined processes. During the study predefined parameters were not monitored and collected on each herd visit, in order to establish a control chart, which could have been used for analysing the data with SPC. Moreover, the key element of implementing alternatives and the subsequent AMU reduction was the coaching of the farmer on a foundation of trust, which was not measurable. Thus, the most challenging and time-consuming part of the study was to convince farmers to alter management procedures. Therefore, the effort to convince farmers to take part in both studies was expectably high. Moreover, the participation depended on voluntary basis and maybe these farmers were in general more interested and hence represented the better performing herds or perceived a higher need to reduce AMU (e.g. herds with a high AMU). As a future perspective, applying similar approaches to larger farmer populations is needed.

## Conclusion

A reduction of AMU on herd level was achievable without major impact on pig health. The results of this study demonstrated that the effect of the interventions on the AMU was rather the conglomeration of herd specific alternative measures and cannot be summarized to a single strategy (‘silver bullet’) that would be effective in every farm. Knowledge about risk factors for a higher AMU, benchmarking of AMU on farms, accompanied by a package of herd-specific reduction measures play a substantial role in the challenge to reduce AMU. Tailor-made interventions and a close cooperation with the herd veterinarian showed to be key determinants to jointly address the challenges in the reduction of AMU and prudent use of antimicrobials. To achieve the most substantial effect especially the suckling and weaned pigs should be targeted in priority for interventions. Besides an optimisation of biosecurity practices, a reduction of group treatments via feed and water through improved health and an increased awareness of treatment and care for individual animals should be included in possible intervention strategies.

## Supplementary information

**Additional file 1.** Details on recruitment of farms.

**Additional file 2.** Distribution of treatment incidences of farrow-to finish pig herds before and after the intervention study.

## Data Availability

The datasets used and analysed during the current study are available from the corresponding author on reasonable request. The dataset supporting the conclusions of this article is included within the article and its additional files.

## Abbreviations

AGP: Antimicrobial growth promotors
AMR: Antimicrobial resistance
AMU: Antimicrobial usage
DDDA: Defined Daily Dose Animal
e.g.: Exempli gratia (for example)
EFSA: European Food Safety Authority
EMA: European Medicines Agency
ESVAC: European Surveillance Veterinary Antimicrobial Consumption
EU: European Union
i.e.: Id est
kg: Kilogram
mg: Milligram
NPD: Neonatal porcine diarrhoea
PEDV: Porcine Epidemic Diarrhoea Virus
ppm: Parts per million
PRRSV: Porcine Respiratory and Reproductive Syndrome Virus
PWD: Post weaning diarrhoea
*S.suis*: *Streptococcus suis*
SPC: Statistical process control
TI: Treatment incidence
