# Supplementary material for: Effectiveness of alternative measures to reduce antimicrobial usage in pig production in four European countries
Source: Porcine Health Manag. 2020 Mar 2;6:6. doi: 10.1186/s40813-020-0145-6 (PMC7050127; doi:10.1186/s40813-020-0145-6)
Supplement: Supplementary file 1 — Additional file 1. Details on recruitment of farms. [file 40813_2020_145_MOESM1_ESM.docx]

**Additional file 1** Details on recruitment of farms

In Belgium a total of 47 herds participated in the cross-sectional study and 29 herds were actively asked to take part in the subsequent intervention study. Sixteen Belgian herds agreed on participation in the intervention study, but one herd was lost due to incomplete follow-up. Based on the results from the cross-sectional study, 30 French farms with the highest AMU were asked to participate in the intervention study and their herd veterinarians were contacted. In case of interest to participate the veterinarians asked the farmers if they were willing to enrol in the study, which 14 farms did. Based on volunteer participation, six additional farms, were recruited via their herd veterinarians, who had already agreed to be part of the intervention study. Unfortunately, one French herd was lost to follow-up. In Germany, all farms which were involved in the cross-sectional study (n=60) were invited to take part in the intervention study. Nineteen farms agreed to participate, and six additional farms were recruited via two veterinary practices who contacted interested farmers. Thus 25 German farms participated in the intervention study. Swedish farmers were approached in a similar way. During the herd visit of the cross-sectional study they were informed about the intervention study and asked to participate. Six farms agreed to enrol in the study. With the aid of the herd veterinarians from the Farm & Animal Health organization (Gård och Djurhälsan (G&D) formerly Swedish Animal Health Service) three additional farms fulfilling the inclusion criteria were recruited. In Germany and Sweden, farmers received financial compensation for collecting and providing data, 200€ and 1300€, respectively.
