# Supplementary material for: Effectiveness of alternative measures to reduce antimicrobial usage in pig production in four European countries
Source: Porcine Health Manag. 2020 Mar 2;6:6. doi: 10.1186/s40813-020-0145-6 (PMC7050127; doi:10.1186/s40813-020-0145-6)
Supplement: Supplementary file 2 — Additional file 2. Distribution of treatment incidences of farrow-to finish pig herds before and after the intervention study. [file 40813_2020_145_MOESM2_ESM.docx]

**Additional file 2** Distribution of treatment incidences of farrow-to finish pig herds before and after the intervention study.

|  | Belgium (n=14) |  | France (n=19) |  | Germany (n=25) |  | Sweden (n=9) |  |
| --- | --- | --- | --- | --- | --- | --- | --- | --- |
|  | Before intervention | After intervention | Before intervention | After intervention | Before intervention | After intervention | Before intervention | After intervention |
| Treatment incidence^a^ | Median  (Q25; Q75) | Median  (Q25; Q75) | Median  (Q25; Q75) | Median  (Q25; Q75) | Median  (Q25; Q75) | Median  (Q25; Q75) | Median  (Q25; Q75) | Median  (Q25; Q75) |
| Age category |  |  |  |  |  |  |  |  |
| Suckling pigs | 315.7  (158.9; 576.4) | 290.7  (116.7; 407.1) | 239.5  (121.3; 435.3) | 155.8  (66.9; 259.9) | 356.4  (248.9; 695.5) | 220.4  (82.5; 809.4) | 160.0  (93.0; 269.5) | 99.0  (58.5; 153.1) |
| Weaned pigs | 168.9  (61.3; 1079.5) | 220.1  (115.1; 573.5) | 1048.3  (756.1; 1358.8) | 516.9  (168.4; 800.9) | 559.6  (257.8; 997.3) | 285.5  (164.6; 572.1) | 11.9  (8.8; 96.3) | 3.8  (2.6; 27.0) |
| Fattening pigs | 6.9  (1.5; 20.8) | 15.0  (5.8; 32.2) | 8.1  (0.2; 35.1) | 2.8  (0.2; 12.7) | 18.9  (0.8; 67.2) | 39.8  (0.4; 75.4) | 5.9  (2.8; 8.7) | 1.7  (0.9; 3.4) |
| TI200d | 118.2  (64.1; 380.1) | 85.2  (57.8; 280.6) | 405.9  (261.6; 813.1) | 240.9  (69.9; 440.8) | 332.2  (133.4; 453.3) | 204.3  (115.4; 293.3) | 35.9  (22.9; 90.9) | 24.5  (17.4; 49.3) |
| Breeding pigs  (Sows, gilts, boars) | 18.4  (0.8; 20.1) | 2.8  (1.9; 18.7) | 22.1  (11.2; 112.6) | 19.5  (6.7; 57.8) | 10.3  (4.2; 27.6) | 41.2  (13.7; 62.9) | 11.3  (5.6; 14.4) | 9.4  (4.2; 16.5) |
| Antimicrobial class |  |  |  |  |  |  |  |  |
| 3rd generation cephalosporins | 83.9  (14.2; 417.0) | 0.7  (0.0; 141.6) | 0.0  (0.0; 2.8) | 0.0  (0.0; 0.0) | 0.1  (0.0; 5.3) | 0.4  (0.0; 20.9) | 0.0  (0.0; 0.0) | 0.0  (0.0; 0.0) |
| Aminoglycosides | 0.0  (0.0; 0.2) | 0.0  (0.0; 0.0) | 0.0  (0.0; 29.1) | 0.0  (0.0; 1.0) | 0.0  (0.0; 9.3) | 0.0  (0.0; 7.5) | 0.0  (0.0; 0.0) | 0.0  (0.0; 0.0) |
| Aminopenicillins | 229.9  (146.8; 368.4) | 215.7  (70.1; 686.1) | 52.7  (23.2; 196.9) | 58.6  (17.9; 123.6) | 391.6  (93.4; 572.9) | 333.8  (212.7; 421.7) | 0.4  (0.0; 13.8) | 2.4  (0.0; 29.3) |
| Benzylpenicillins | 0.0  (0.0; 5.2) | 0.0  (0.0; 39.6) | 0.0  (0.0; 0.0) | 0.0  (0.0; 0.0) | 0.0  (0.0; 9.8) | 0.3  (0.0; 7.5) | 107.8  (75.7; 179.2) | 81.4  (33.0; 121.4) |
| Benzylpenicillin-combinations | 0.0  (0.0; 0.0) | 0.0  (0.0; 0.0) | 0.0  (0.0; 18.1) | 0.4  (0.0; 10.6) | 9.9  (0.0; 154.2) | 0.0  (0.0; 43.3) | 0.0  (0.0; 0.0) | 0.0  (0.0; 0.0) |
| Florfenicols | 0.0  (0.0; 0.0) | 0.0  (0.0; 0.0) | 0.1  (0.0; 5.9) | 0.0  (0.0; 11.9) | 0.0  (0.0; 0.0) | 0.0  (0.0; 0.0) | 0.0  (0.0; 0.0) | 0.0  (0.0; 0.0) |
| Fluorquinolones | 16.1  (0.0; 65.9) | 7.6  (3.7; 24.2) | 11.2  (5.3; 90.5) | 18.4  (2.5; 57.4) | 9.6  (5.3; 23.5) | 13.2  (2.7; 22.8) | 0.0  (0.0; 0.0) | 0.0  (0.0; 0.0) |
| Macrolides | 2.5  (0.2; 38.7) | 27.5  (1.7; 261.3) | 18.6  (3.3; 151.5) | 4.7  (0.4; 116.5) | 158.0  (0.6; 448.9) | 131.1  (0.0; 362.5) | 0.0  (0.0; 7.9) | 0.0  (0.0; 0.0) |
| Macrolide-combinations | 0.1  (0.0; 0.6) | 0.0  (0.0; 0.3) | 0.0  (0.0; 1.9) | 0.0  (0.0; 0.5) | 0.0  (0.0; 1.1) | 0.0  (0.0; 0.0) | 0.0  (0.0; 0.0) | 0.0  (0.0; 0.0) |
| Polymyxins | 10.7  (0.0; 111.2) | 14.0  (0.0; 28.9) | 495.0  (429.5; 718.5) | 229.8  (80.2; 408.6) | 97.6  (4.5; 150.2) | 37.4  (0.0; 84.9) | 0.0  (0.0; 10.0) | 0.0  (0.0; 0.0) |
| Pleuromutilins | 0.0  (0.0; 0.0) | 0.0  (0.0; 0.0) | 0.0  (0.0; 0.1) | 0.0  (0.0; 0.0) | 0.0  (0.0; 0.0) | 0.0  (0.0; 0.0) | 0.0  (0.0; 0.0) | 0.0  (0.0; 0.0) |
| Tetracyclines | 17.7  (0.0; 20.5) | 13.8  (5.4; 30.1) | 213.6  (31.3; 397.9) | 27.1  (4.3; 167.2) | 73.7  (16.2; 260.3) | 78.4  (4.5; 205.9) | 0.2  (0.0; 0.3) | 0.2  (0.0; 1.0) |
| Trimethoprim-Sulfonamides | 5.7  (0.0; 26.3) | 0.0  (0.0; 19.1) | 0.0  (0.0; 70.7) | 0.0  (0.0; 46.9) | 1.9  (0.0; 30.9) | 0.1  (0.0; 23.5) | 20.4  (14.3; 28.8) | 7.8  (3.3; 19.8) |
| Administration route |  |  |  |  |  |  |  |  |
| Feed-water | 261.4  (62.6; 1309.2) | 382.6  (120.5; 683.1) | 1223.2  (766.9; 1421.0) | 560.6  (276.0; 872.6) | 573.7  (157.1; 1098.4) | 379.0  (238.8; 633.9) | 0.0  (0.0; 5.6) | 0.0  (0.0; 0.7) |
| Parenteral | 316.9  (168.2; 635.5) | 260.9  (123.3; 412.7) | 248.3  (92.8; 438.6) | 186.0  (90.9; 238.1) | 400.1  (252.5; 719.7) | 325.2  (148.8; 858.6) | 181.0  (119.0; 239.3) | 162.0  (100.6; 177.7) |
| Oral | 0.0  (0.0; 0.3) | 0.0  (0.0; 0.2) | 0.0  (0.0; 0.1) | 0.0  (0.0; 0.0) | 0.0  (0.0; 0.2) | 0.0  (0.0; 0.3) | 0.0  (0.0; 0.0) | 0.0  (0.0; 1.1) |

The TI per 1000 pigs-days at risk is shown for different age categories (including a calculated TI for growing pigs from birth to slaughter with a standardised life span of 200 days), antimicrobial class and administration route. ^a^ For example: a TI of 1000 implies that the animals were treated 1000/1000 days, or 100% of their lifespan/period duration.
